# Patterns of spironolactone management following hyperkalemia among hospitalized patients with heart failure and reduced ejection fraction: a retrospective single-center study

**DOI:** 10.3389/fmed.2026.1870470

**Published:** 2026-08-24

**Authors:** Abdullah Sameer Alshammari, Foud O. Bahamdain, Asma M. Alshahrani, Geetha Kandasamy, Ageeli Rabea Mussa Rabea, Wid Alyamani, Anas Idris, Yara Kamfar, Maher Almalki, Saleh Alghamdi, Mohammed Kanan, Nisrin Bifari, Shaimaa Alsulami, Abdulmalik S. Alotaibi, Emad Elkholy

**Affiliations:** 1 Pharmaceutcial Practices Department, College of Pharmacy, Umm Al-qura University, Makkah, Saudi Arabia; 2 Department of Pharmaceutical Care Services, King Abdullah Medical City, Makkah, Saudi Arabia; 3 Department of Clinical Pharmacy, College of Pharmacy, Shaqra University, Dawadimi, Saudi Arabia; 4 Department of Clinical Pharmacy, College of Pharmacy, King Khalid University, Abha, Saudi Arabia; 5 Pharmaceutical Services Administration, King Faisal Hospital-Holy Capital Makkah, Makkah, Saudi Arabia; 6 Nahdi Medical Company, Makkah, Saudi Arabia; 7 Department of Clinical Pharmacy, Faculty of Pharmacy, Al-Baha University, Al-Baha, Saudi Arabia; 8 Pharmaceutical Care Administration, Rafha General Hospital, Northern Border Health Cluster, Rafha, Saudi Arabia

**Keywords:** chronic kidney disease, clinical outcomes, guideline-directed medical therapy, heart failure reduced ejection fraction, hyperkalemia, retrospective study, spironolactone

## Abstract

**Background:**

Heart failure with reduced ejection fraction (HFrEF) is a major cause of morbidity and mortality worldwide. Hyperkalemia is a common complication of chronic spironolactone therapy and may necessitate dose reduction or discontinuation, particularly in patients with impaired renal function. This study aimed to describe real-world spironolactone management patterns following the occurrence of hyperkalemia, evaluate guideline-directed medical therapy (GDMT) exposure (receipt of ≥3 drug classes), and explore factors associated with in-hospital mortality among hospitalized patients with HFrEF and hyperkalemia receiving chronic spironolactone therapy.

**Methods:**

This retrospective single-center study included 146 adult HFrEF patients on spironolactone (May–Dec 2023). Data on demographics, comorbidities, renal function, GDMT exposure, and outcomes were extracted from electronic records. Hyperkalemia was categorized as mild–moderate (5.0–5.5 mmol/L) or severe (>5.5 mmol/L). Univariate logistic regression and multivariable Cox models identified predictors of in-hospital mortality. Kaplan–Meier analysis compared survival by GDMT exposure.

**Results:**

The study cohort (*N* = 146) had a mean age of 58.34 ± 12.89 years, with 74% having hypertension and 23% having chronic kidney disease. Among the included HFrEF patients receiving chronic spironolactone therapy and admitted with hyperkalemia, 87% had mild–moderate and 13% had severe hyperkalemia at admission. Among the 136 patients with documented spironolactone management decisions, therapy was continued at the same dose in 13.2% of patients, dose reduced in 28.7%, and discontinued in 58.1%. Among patients with available follow-up potassium measurements, continuation of spironolactone at the same dose and dose reduction were associated with significant reductions in serum potassium levels over 72 h (*p* = 0.04 and *p* < 0.001, respectively), whereas discontinuation showed no significant change (*p* = 0.18). In exploratory multivariable Cox regression analysis, increasing age (HR 1.05, 95% CI 1.01–1.10, *p* = 0.02) and chronic kidney disease (HR 2.10, 95% CI 1.05–4.20, *p* = 0.03) were associated with higher in-hospital mortality. NYHA class III–IV showed a higher hazard estimate but did not reach statistical significance (HR 1.55, 95% CI 0.95–2.80, *p* = 0.08). In a separate exploratory adjusted analysis, receipt of ≥3 GDMT classes yielded a lower hazard estimate for in-hospital mortality (adjusted HR 0.62, 95% CI 0.21–1.80); however, this association did not reach statistical significance (*p* = 0.30).

**Conclusions:**

In this retrospective cohort of hospitalized patients with HFrEF and hyperkalemia, spironolactone management most commonly involved dose reduction or discontinuation following hyperkalemia. Older age and chronic kidney disease were associated with higher in-hospital mortality in exploratory analyses, whereas receipt of ≥3 GDMT classes was associated with a lower adjusted hazard estimate that did not reach statistical significance. Overall, these findings are exploratory and hypothesis-generating and should be interpreted cautiously given the retrospective single-center design, the limited number of mortality events, and the potential for residual confounding.

## 1 Introduction

Heart failure with reduced ejection fraction (HFrEF) is a prevalent cardiovascular condition marked by impaired systolic function, where the heart is unable to pump blood effectively (1). This results in reduced functional capacity and impaired quality of life (2). HFrEF is a leading cause of morbidity, mortality, and hospitalization worldwide, particularly in older populations with concomitant comorbidities such as hypertension, diabetes, and coronary artery disease (3). Globally, heart failure affects over 26 million people, with increasing prevalence and associated healthcare costs, highlighting its major public health impact (4, 5). Guideline-directed medical therapy (GDMT) has substantially improved clinical outcomes in patients with HFrEF and includes angiotensin-converting enzyme inhibitors (ACEIs), angiotensin receptor blockers (ARBs), beta-blockers, and mineralocorticoid receptor antagonists (MRAs) (6).

Spironolactone is an aldosterone antagonist that has demonstrated significant benefits in improving both survival and symptom control in patients with HFrEF (7). Its therapeutic effects are mediated through blockade of aldosterone, reducing sodium retention and adverse cardiac remodeling (8, 9). The efficacy of spironolactone in improving clinical outcomes, particularly in patients with severe heart failure, was demonstrated in large clinical trials such as the RALES (randomized aldactone evaluation study), where it was shown to reduce mortality and hospitalizations in patients with severe, symptomatic heart failure (10). Mineralocorticoid receptor antagonists (MRAs), particularly spironolactone, remain a cornerstone of GDMT for patients with HFrEF. Recent consensus guidelines from the 2024 American College of Cardiology (ACC) emphasize that MRAs should be incorporated as part of comprehensive guideline-directed medical therapy to improve clinical outcomes in this population (11). In a U.S. retrospective cohort study, only about 20% of patients with HFrEF were prescribed spironolactone, indicating under-utilization of this therapy in routine clinical practice despite guideline recommendations (12). These findings underscore the importance of evaluating spironolactone use, adherence to GDMT, and associated clinical outcomes in real-world clinical settings.

However, data from routine clinical practice often differ from randomized clinical trials, and real-world evidence regarding spironolactone use, safety, and adherence to guideline-directed medical therapy remains limited, particularly in single-center settings (13, 14). Additionally, patterns of receipt of GDMT and factors influencing spironolactone management in routine clinical practice remain incompletely characterized (15). Hyperkalemia is a known side effect of spironolactone, especially in patients with renal dysfunction or those receiving other medications that affect potassium balance (16). Given the risks associated with hyperkalemia, careful monitoring of potassium levels is essential for patients on spironolactone (17). This study aimed to describe real-world spironolactone management patterns following the occurrence of hyperkalemia, evaluate guideline-directed medical therapy (GDMT) exposure (receipt of ≥3 drug classes), and explore factors associated with in-hospital mortality among hospitalized patients with HFrEF and hyperkalemia receiving chronic spironolactone therapy.

## 2 Methods

### 2.1 Study design and setting

This was a retrospective, observational, single-center study conducted at a tertiary-care hospital with a dedicated heart failure clinic in Saudi Arabia from May 2023 to December 2023. The study evaluated spironolactone management strategies in patients with heart failure with reduced ejection fraction (HFrEF). The study cohort consisted of patients with HFrEF who were receiving chronic spironolactone therapy and were admitted with hyperkalemia during the index hospitalization. The analysis focused on treatment decisions following detection of hyperkalemia and associated changes in serum potassium levels rather than the incidence of hyperkalemia.

### 2.2 Sample size determination

**Figure 1:**
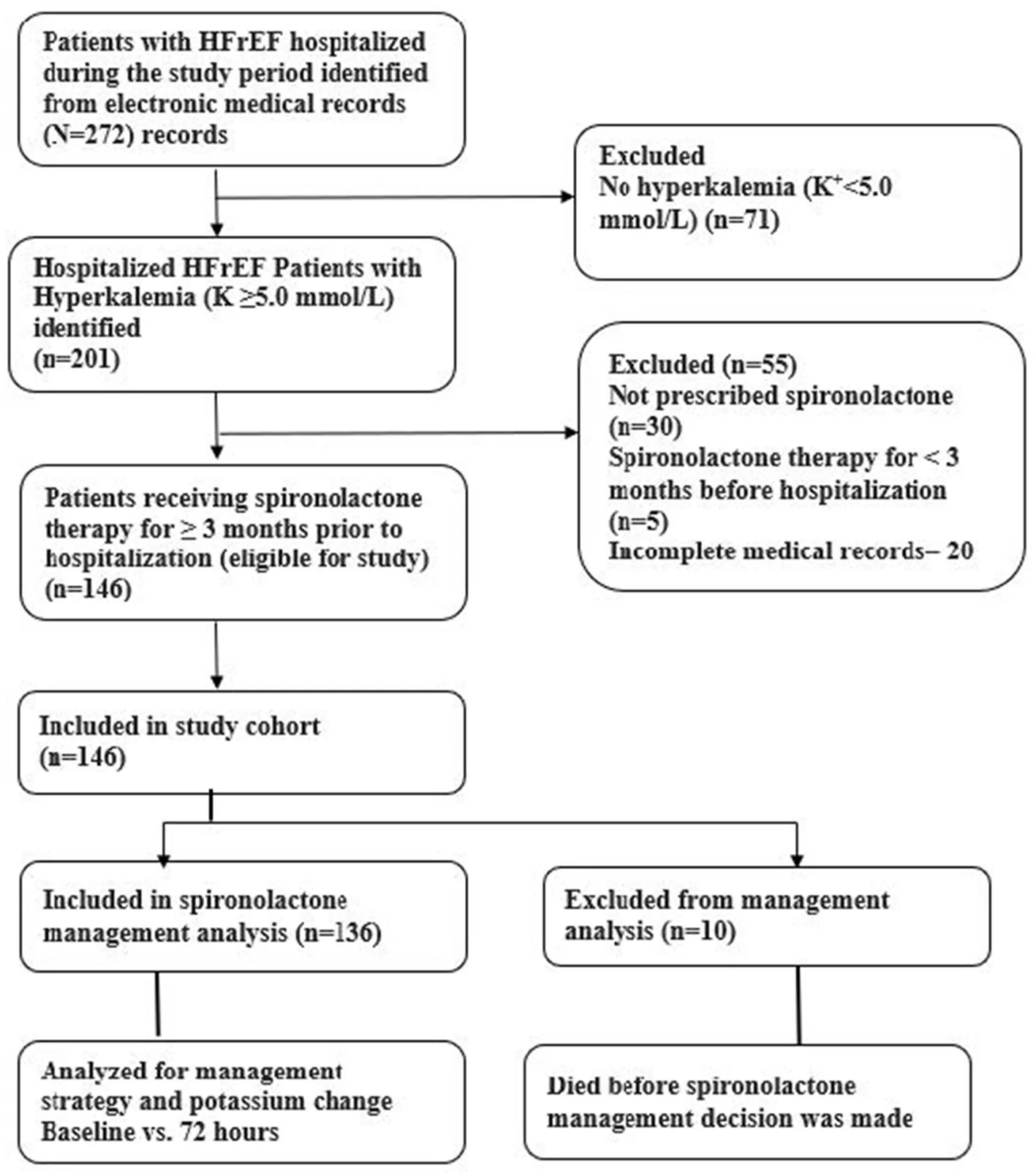
Flow diagram of patient selection and inclusion in the retrospective study cohort of hospitalized HFrEF patients with hyperkalemia.

As this was a retrospective observational study, the sample size was determined based on the number of eligible patients available in the electronic medical record (EHR) database during the predefined study period rather than through an *a priori* power calculation. All consecutive adult patients with HFrEF receiving chronic spironolactone therapy and admitted with hyperkalemia (serum potassium >5.0 mmol/L) between May 2023 and December 2023 were screened. A total of 272 hospitalized HFrEF patients were identified, of whom 146 met the predefined eligibility criteria and were included in the final study cohort. The patient selection process is illustrated in Figure 1.

### 2.3 Inclusion and exclusion criteria

As this was a retrospective study, the diagnosis of HFrEF was based on documentation in the patients' electronic health records (EHR) by the treating physician, supported by echocardiographic confirmation of left ventricular ejection fraction (LVEF) ≤ 40%.

#### 2.3.1 Inclusion criteria

Adult patients (≥18 years) with heart failure with reduced ejection fraction (HFrEF) who were on chronic spironolactone therapy for at least 3 months and were admitted with hyperkalemia (serum potassium >5.0 mmol/L) during the index hospitalization were included.

#### 2.3.2 Exclusion criteria

Patients were excluded if they had absolute contraindications to spironolactone, such as severe renal impairment (serum creatinine >2.5 mg/dl or end-stage renal disease), a known hypersensitivity to mineralocorticoid receptor antagonists, or incomplete clinical or laboratory records relevant to potassium monitoring and spironolactone therapy.

### 2.4 Data collection

All eligible patients were identified from the EHR system. Data were collected retrospectively for patients with HFrEF who had been on chronic spironolactone therapy for at least 3 months prior to admission. Collected information included demographic data (age, sex, BMI, and comorbidities such as hypertension, diabetes, and coronary artery disease), clinical characteristics (ejection fraction, New York Heart Association [NYHA] functional class, and relevant medical history), and laboratory values, including serum potassium concentrations obtained from routine venous blood samples recorded in the electronic health record (mmol/L) and serum creatinine (mg/dL) and estimated glomerular filtration rate (eGFR expressed as ml/min/1.73m^2^). The eGFR values were obtained from the electronic health record laboratory system and were calculated using the chronic kidney disease epidemiology collaboration (CKD-EPI) equation based on serum creatinine, age, and sex.

Medication data were extracted from the electronic prescribing system, including angiotensin-converting enzyme inhibitors (ACEIs), angiotensin receptor blockers (ARBs), angiotensin receptor–neprilysin inhibitors (ARNIs), beta-blockers, and sodium-glucose co-transporter-2 (SGLT2) inhibitors. Mineralocorticoid receptor antagonists (MRAs) were not included in the GDMT exposure definition because all patients in this cohort were already receiving chronic spironolactone therapy at baseline. Receipt of ≥3 GDMT classes was defined as prescription of at least three guideline-recommended HFrEF medication classes (ACEI/ARB/ARNI, beta-blocker, and SGLT2 inhibitor). This variable was considered a measure of prescribed medication class exposure rather than a direct measure of medication adherence or treatment optimization.

Chronic kidney disease (CKD) was defined based on documented clinician diagnosis recorded in the electronic health record (EHR) during the index hospitalization. Baseline potassium was defined as serum potassium at hospital admission. Hyperkalemia was defined as a serum potassium level >5.0 mmol/L and classified as mild–moderate (5.0–5.5 mmol/L) or severe (>5.5 mmol/L) (18). Follow-up serum potassium values were extracted from routine laboratory measurements documented in the electronic health record and recorded approximately 72 h after the spironolactone management decision (continuation at the same dose, dose reduction, or discontinuation), according to routine inpatient clinical care. The timing and frequency of potassium measurements were determined by the treating physicians according to routine clinical practice and individual patient needs rather than a predefined study-specific protocol. Follow-up serum potassium measurements were available for all patients included in the analytic cohort (*n* = 136). 10 patients who died before spironolactone dose adjustment did not have 72-h potassium measurements and were therefore excluded from the longitudinal potassium analysis. Clinical outcomes included in-hospital mortality and changes in serum potassium associated with the observed spironolactone management patterns.

### 2.5 Statistical analysis

Statistical analyses were conducted using SPSS version 28. Continuous data are summarized as mean ± standard deviation, while categorical data are expressed as counts and percentages. Missing data were assessed during data extraction. No imputation methods were applied, and analyses were performed using available data. Unadjusted associations with in-hospital mortality were examined using binary logistic regression. Given the limited number of mortality events, mortality analyses were considered exploratory. A limited-variable Cox proportional hazards model including clinically relevant covariates was used to evaluate factors associated with time to in-hospital mortality. The Cox model was selected because it allowed incorporation of the timing of in-hospital mortality events and differences in observation duration, with patients discharged alive treated as censored observations.

For time-to-event analyses, time zero (t0) was defined as the date of admission for the index hospitalization during which hyperkalemia was identified. The event of interest was in-hospital mortality, and event time was calculated as the number of days from hospital admission to death. Patients discharged alive were administratively censored at the date of discharge; therefore, length of stay represented the follow-up duration for censored individuals. Because follow-up was restricted to the index hospitalization and outcomes were obtained from the electronic health record, there was no loss to follow-up. The proportional hazards assumption was assessed using graphical inspection of log-minus-log survival plots. No major violations were identified; however, the results were interpreted cautiously given the limited number of mortality events (*n* = 10). Changes in serum potassium from baseline to 72 h within each spironolactone management group were analyzed using paired *t*-tests. Kaplan–Meier survival curves were generated according to receipt of ≥3 GDMT classes and compared using the log-rank test. Spironolactone management strategy after hyperkalemia identification was not included as a baseline exposure in survival analyses because treatment modification occurred after admission and could introduce immortal-time and reverse-causation bias. Patients who died before a spironolactone management decision was made were not assigned to a spironolactone management category but remained included in the overall in-hospital mortality analysis. Statistical significance was defined as *p* < 0.05.

### 2.6 Ethical approval

This study was reviewed and approved by the Institutional Ethics Committee at King Abdulaziz Medical City (IRB No. 23-1067). All patient data were handled confidentially, and personal identifiers were anonymized to ensure privacy. Given the retrospective nature of the study and use of anonymized electronic health record data, individual patient informed consent was waived by the Institutional Ethics Committee.

## 3 Results

A total of 146 patients with heart failure with reduced ejection fraction were included in the study. The cohort was predominantly male (84.3%), with a mean age of 58.3 ± 12.9 years and a mean body mass index of 27.4 ± 5.9 kg/m^2^. The mean left ventricular ejection fraction was 25.1 ± 7.8%, consistent with reduced systolic function. Most patients were classified as NYHA class III or IV (69.2%). Comorbid conditions were common, with diabetes mellitus (77.4%) and hypertension (74.0%) being the most prevalent, followed by myocardial infarction (30.1%), chronic kidney disease (22.6%), and stroke (11.6%).

Regarding pharmacological management, beta-blockers were used in the majority of patients (94.5%), while ACE inhibitors/angiotensin receptor blockers were prescribed in 43.8% and angiotensin receptor–neprilysin inhibitors in 25.3%. All patients received a mineralocorticoid receptor antagonist (spironolactone) as per the study inclusion criteria. SGLT2 inhibitors were used in 42.5% of patients. Overall, 39.72% of patients received ≥3 GDMT medication classes, defined as exposure to three or more guideline-recommended HFrEF medication classes (Table 1).

**Table 1:** Baseline demographic, clinical characteristics, and guideline-directed medical therapy (GDMT).

Variable	Total cohort (*N* = 146)
Demographics	
Male *n* (%)	123 (84.25)
Female n (%)	23 (15.75)
Age (years), mean ± SD	58.34 ± 12.89
Body mass index (kg/m^2^), mean ± SD	27.35 ± 5.91
Heart failure severity	
Left ventricular ejection fraction (%), mean ± SD	25.12 ± 7.82
NYHA class I, *n* (%)	18 (12.33)
NYHA class II, *n* (%)	27 (18.49)
NYHA class III, *n* (%)	72 (49.32)
NYHA class IV, *n* (%)	29 (19.86)
Comorbidities	
Hypertension, *n* (%)	108 (73.97)
Diabetes mellitus, *n* (%)	113 (77.40)
Myocardial infarction, *n* (%)	44 (30.14)
Chronic kidney disease, *n* (%)	33 (22.60)
Stroke, *n* (%)	17 (11.64)
Beta-blockers, *n* (%)	138 (94.52)
ACE inhibitors/ARBs, *n* (%)	64 (43.84)
ARNI, *n* (%)	37 (25.34)
Mineralocorticoid receptor antagonist (spironolactone), *n* (%)	146 (100.00)
SGLT2 inhibitors, *n* (%)	62 (42.47)
Receipt of ≥3 GDMT classes	58 (39.72)

The mean serum creatinine level was 1.33 ± 0.46 mg/dl, with a mean eGFR of 70.1 ± 29.5 ml/min/1.73m^2^. The mean blood urea nitrogen level was 28.2 ± 15.4 mg/dl. The average serum potassium concentration was 5.33 ± 0.28 mmol/L, consistent with the hyperkalemia criteria used for study inclusion among patients receiving mineralocorticoid receptor antagonist therapy (Table 2).

**Table 2:** Baseline renal function and laboratory parameters.

Parameter	Mean ±SD
Serum creatinine (mg/dl)	1.33 ± 0.46
eGFR (ml/min/1.73m^2^)	70.05 ± 29.51
Blood urea nitrogen (mg/dl)	28.20 ± 15.41
Serum potassium (mmol/L)	5.33 ± 0.28

When stratified by hyperkalemia severity, spironolactone management strategies were significantly different between patients with mild–moderate and severe hyperkalemia (Table 3). Among patients with mild–moderate hyperkalemia (*n* = 117), spironolactone was continued at the same dose in 18 patients (15.4%), dose reduced in 37 patients (31.6%), and discontinued in 62 patients (53.0%). Among patients with severe hyperkalemia (*n* = 19), spironolactone was discontinued in 17 patients (89.5%) and dose reduced in 2 patients (10.5%), with no patients continuing the same dose. Overall, among the 136 patients with documented spironolactone management decisions, therapy was continued at the same dose in 18 patients (13.2%), dose reduced in 39 patients (28.7%), and discontinued in 79 patients (58.1%). The distribution of management strategies differed significantly according to hyperkalemia severity (Fisher's exact test, *p* = 0.002). 10 patients who died before spironolactone management decisions were made were excluded from the management strategy analysis (Table 3). Baseline spironolactone dose according to subsequent management strategy is presented in Supplementary Table 2. Higher baseline spironolactone doses were more frequently associated with dose reduction or discontinuation compared with continuation of therapy (*p* < 0.001).

**Table 3:** Subgroup analysis of spironolactone management strategy according to hyperkalemia severity.

Hyperkalemia severity	Continued at same dose, *n* (%)	Dose reduced, *n* (%)	Discontinued, *n* (%)	Total, *n* (%)	*P*-value
Mild–moderate hyperkalemia (5.0–5.5 mmol/L)	18 (15.38)	37 (31.62)	62 (52.99)	117 (100.00)	–
Severe hyperkalemia (>5.5 mmol/L)	0 (0.00)	2 (10.53)	17 (89.47)	19 (100.00)	–
Total analyzed	18 (13.24)	39 (28.68)	79 (58.09)	136 (100.00)	0.002^*^

^*^P-value calculated using Fisher's exact test. 10 patients died before spironolactone management decisions were made and were not included in management strategy calculations.

Changes in serum potassium levels according to spironolactone management strategy are presented in Table 4. Among patients who continued spironolactone at the same dose, mean serum potassium decreased from 5.25 ± 0.30 mmol/L at baseline to 5.05 ± 0.26 mmol/L at 72 h, with a mean change of −0.20 mmol/L (95% CI −0.38 to −0.02; *p* = 0.04). Patients with dose reduction showed a greater mean decrease in serum potassium, from 5.37 ± 0.32 mmol/L to 4.92 ± 0.29 mmol/L (mean change −0.45 mmol/L; 95% CI −0.58 to −0.32; *p* < 0.001). In patients where spironolactone was discontinued, serum potassium changed from 5.27 ± 0.28 mmol/L to 5.22 ± 0.27 mmol/L (mean change −0.05 mmol/L; 95% CI −0.14–0.04; *p* = 0.18). The between-group comparison of mean potassium changes demonstrated a statistically significant difference among management groups (one-way ANOVA, *p* = 0.003). 10 patients who died before spironolactone dose adjustment were excluded from this analysis because 72-h potassium measurements and management data were unavailable.

**Table 4:** Serum potassium at baseline and 72 h by spironolactone management strategy.

Spironolactone management strategy	*n*	Baseline K^+^ (mmol/L) Mean ±SD	72-h K^+^ (mmol/L) Mean ±SD	Mean change (ΔK^+^)	95% CI	*p*-value
Continued at same dose	18	5.25 ± 0.30	5.05 ± 0.26	−0.20	−0.38 to −0.02	0.04
Dose reduced	39	5.37 ± 0.32	4.92 ± 0.29	−0.45	−0.58 to −0.32	< 0.001
Discontinued	79	5.27 ± 0.28	5.22 ± 0.27	−0.05	−0.14 to 0.04	0.18

Between-group comparison of ΔK^+^: *p* = 0.003 – using oneway ANOVA. 10 patients who died before spironolactone dose adjustment were excluded from this analysis because 72-h potassium measurements and treatment modification data were unavailable. Percentages were calculated using the analytic cohort (*N* = 136).

Unadjusted associations between clinical variables and in-hospital mortality are presented in Table 5. Increasing age was associated with higher odds of in-hospital mortality (OR 1.07 per year increase, 95% CI 1.02–1.13; *p* = 0.01). Chronic kidney disease also showed higher unadjusted odds of in-hospital mortality (OR 3.86, 95% CI 1.00–14.90; *p* = 0.05). No statistically significant unadjusted associations were observed for sex (OR 0.58, 95% CI 0.07–4.70; *p* = 0.60), NYHA class III–IV (OR 1.85, 95% CI 0.40–8.40; *p* = 0.39), severe hyperkalemia (OR 0.73, 95% CI 0.08–6.50; *p* = 0.77), SGLT2 inhibitor use (OR 0.32, 95% CI 0.07–1.50; *p* = 0.15), or receipt of ≥3 GDMT medication classes (OR 1.01, 95% CI 0.27–3.76; *p* = 0.99).

**Table 5:** Unadjusted association between clinical variables and in-hospital mortality.

Variable	Death *n/N* (%)	Survival *n/N* (%)	Unadjusted OR (95% CI)	*p*-value
Age (per year increase)	—	—	1.07 (1.02–1.13)	0.01
Sex (female vs. male)	1/23 (4.35) vs. 9/123 (7.32	22/23 (95.65) vs. 114/123 (92.68)	0.58 (0.07–4.70)	0.60
NYHA class III–IV	8/101 (7.92)	93/101 (92.08)	1.85 (0.40–8.40)	0.39
Chronic kidney disease	5/33 (15.15)	28/33 (84.85)	3.86 (1.00–14.90)	0.05
Severe hyperkalemia	1/19 (5.26)	18/19 (94.74)	0.73 (0.08–6.50)	0.77
SGLT2 inhibitor use	2/62 (3.23)	60/62 (96.77)	0.32 (0.07–1.50)	0.15
Receipt of ≥3 GDMT medication classes	4/58 (6.89)	54/58 (93.11)	1.01 (0.27–3.76)	0.99

An exploratory Cox proportional hazards regression analysis was performed to identify factors associated with in-hospital mortality (Table 6). Due to the limited number of mortality events (*n* = 10), a limited-variable model was used, and the findings were interpreted cautiously. Increasing age was associated with a higher hazard of in-hospital mortality (adjusted HR 1.05; 95% CI 1.01–1.10; *p* = 0.02). Chronic kidney disease was also associated with a higher hazard of mortality (adjusted HR 2.10; 95% CI 1.05–4.20; *p* = 0.03). NYHA class III–IV showed a higher hazard estimate; however, the association did not reach statistical significance (adjusted HR 1.55; 95% CI 0.95–2.80; *p* = 0.08).

**Table 6:** Exploratory cox proportional hazards regression analysis of factors associated with in-hospital mortality.

Variable	Adjusted HR	95% CI	*p*-value
Age (per year increase)	1.05	1.01–1.10	0.02
Chronic kidney disease	2.10	1.05–4.20	0.03
NYHA class III–IV	1.55	0.95–2.80	0.08

Due to the limited number of mortality events (*n* = 10), a limited-variable exploratory model was used. Findings should be interpreted cautiously.

The association between receipt of ≥3 GDMT medication classes and in-hospital mortality is presented in Table 7. The association was evaluated using an exploratory adjusted Cox regression model. Among patients receiving fewer than three GDMT classes, 6 of 88 (6.81%) died during hospitalization, compared with 4 of 58 (6.89%) among those receiving ≥3 GDMT classes. After adjustment for age, chronic kidney disease, and NYHA functional class III–IV, receipt of ≥3 GDMT classes was associated with a lower hazard estimate for in-hospital mortality (adjusted HR 0.62, 95% CI 0.21–1.80); however, this association did not reach statistical significance (*p* = 0.30).

**Table 7:** Receipt of ≥3 guideline-directed medical therapy (GDMT) classes and in-hospital mortality.

GDMT group	Mortality *n* (%)	Adjusted HR (95% CI)	*p*-value
< 3 classes (*n* = 88)	6 (6.81)	Reference	—
≥3 classes (*n* = 58)	4 (6.89)	0.62 (95% CI 0.21–1.80)	0.30

The adjusted model evaluating GDMT exposure included the following covariates: age, chronic kidney disease (CKD), and NYHA functional class III–IV. These covariates were selected based on their clinical relevance while limiting the number of variables included in the model because of the small number of mortality events.

Given the limited number of mortality events, these findings are presented as exploratory.

**Figure 2:**
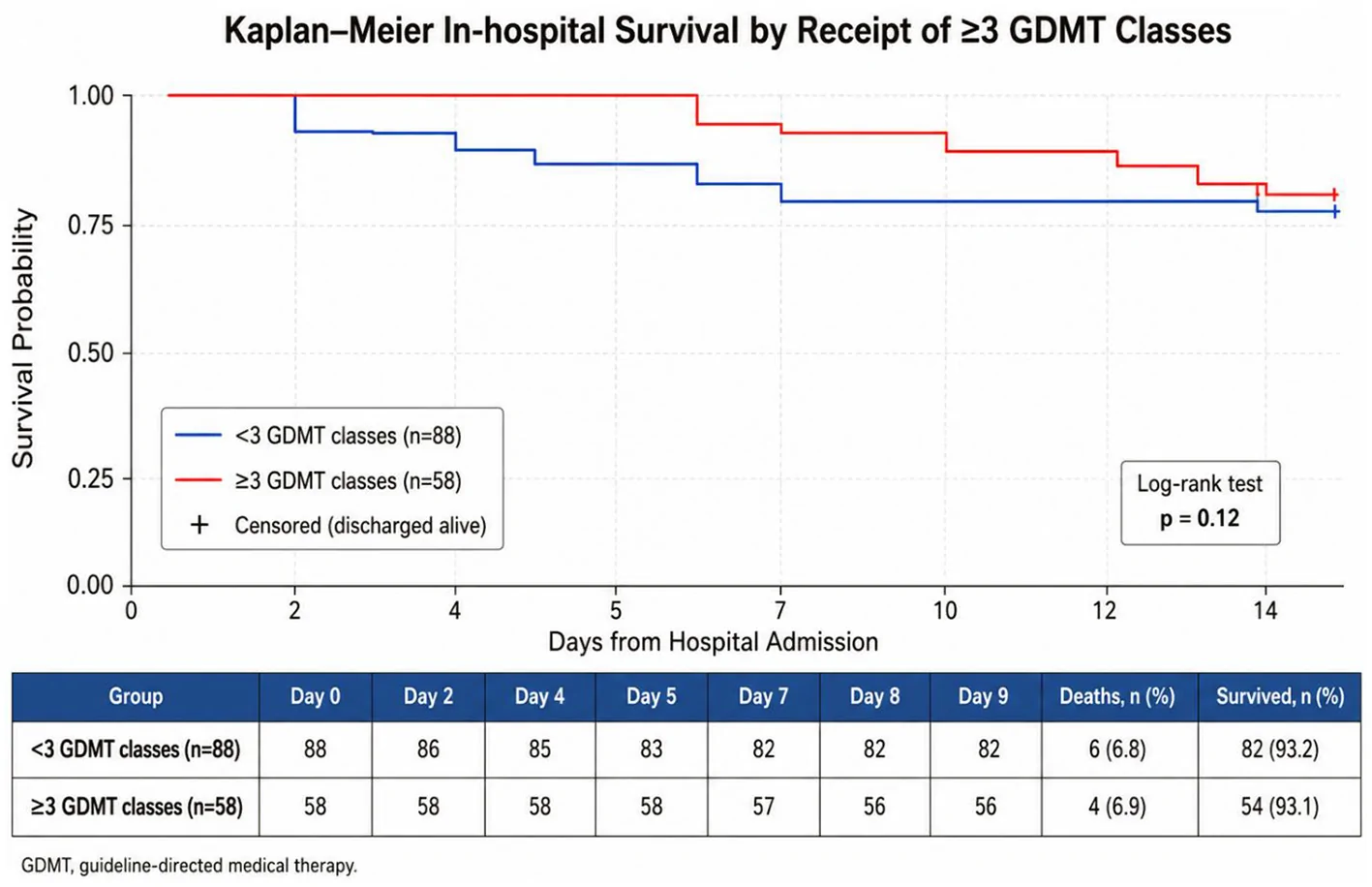
Survival analysis vs. Guideline-directed medical therapy. Log rank *P* = 0.12.

Kaplan–Meier survival analysis showed a higher observed in-hospital survival probability among patients receiving ≥3 GDMT medication classes compared with those receiving < 3 classes (Figure 2). However, the difference between the survival curves was not statistically significant (log-rank *p* = 0.12).

## 4 Discussion

This retrospective study describes real-world spironolactone management patterns following hyperkalemia and explores associated short-term clinical outcomes among hospitalized patients with HFrEF receiving chronic spironolactone therapy. The demographic characteristics observed in this cohort are consistent with previous reports indicating higher prevalence and earlier onset of heart failure among men (19, 20). The burden of cardiovascular comorbidities observed in this cohort reflects trends reported in large epidemiological studies, including the Framingham Heart Study (21). The pharmacotherapy profile reflected partial implementation of guideline-directed medical therapy. Beta-blocker therapy was broadly consistent with guideline recommendations (22, 23), whereas implementation of other components of GDMT appeared less comprehensive. Although this pattern may suggest potential gaps in comprehensive GDMT implementation, it should be interpreted in the context of a hospitalized HFrEF population, where factors such as renal dysfunction, hyperkalemia risk, hypotension, intolerance, or recent clinical instability may influence prescribing practices. Real-world evidence indicates that GDMT is often under-prescribed and not titrated to guideline-recommended target doses, particularly among older adults and patients with comorbidities (14). The 2023 ESC Focused Update reinforces the importance of implementing comprehensive GDMT, including RAAS inhibitors, MRAs, and SGLT2 inhibitors, in eligible patients with HFrEF while considering individual patient characteristics and treatment tolerability (24). These findings emphasize the importance of optimizing guideline-directed medical therapy whenever clinically feasible while balancing established prognostic benefits against patient-specific risks, consistent with landmark trials and contemporary guidelines (24–26).

Spironolactone-associated hyperkalemia remains an important safety consideration, particularly among patients with impaired renal function or those receiving concomitant potassium-raising therapies (26). Management decisions require careful monitoring and individualized adjustment according to clinical context. Real-world data have shown that spironolactone, especially when used alongside ACE inhibitors or ARBs, may increase the likelihood of hyperkalemia (27). Additionally, other studies have reported hyperkalemia as a notable safety consideration during spironolactone therapy (28), and meta-analyses have confirmed hyperkalemia as a frequent adverse effect of mineralocorticoid receptor antagonists in heart failure (29).

Chronic kidney disease was frequently observed in this cohort, consistent with findings from other heart failure populations. CKD is recognized as a common comorbidity in heart failure and is associated with an elevated risk of electrolyte disturbances during MRA therapy (30). The KDIGO 2024 CKD guideline emphasizes monitoring kidney function and serum potassium in patients receiving RAAS inhibitors, particularly among individuals with CKD who have an increased susceptibility to hyperkalemia (31). A prior study by Zhang et al. evaluated the safety of spironolactone in patients with CKD and emphasized the importance of monitoring potassium and renal function during therapy (32).

Regarding treatment adjustments, dose reduction and discontinuation were the most common spironolactone management strategies following hyperkalemia in hospitalized patients with HFrEF. The need for these modifications may reflect the clinical complexity of this population, where renal dysfunction, hyperkalemia risk, and overall illness severity influence treatment decisions. These patterns align with real-world evidence showing that hyperkalemia and renal dysfunction are common reasons for MRA dose modification or discontinuation (33). Spironolactone discontinuation in this setting highlights the clinical challenge of balancing safety concerns with the established benefits of MRA therapy. Previous studies have shown that discontinuation of mineralocorticoid receptor antagonist therapy is common in real-world HFrEF populations and has been reported in association with poorer clinical outcomes (34).

However, contemporary recommendations emphasize active management of hyperkalemia to avoid unnecessary discontinuation of prognostically beneficial RAAS inhibitor therapy. Recommended approaches include identification and correction of reversible contributors, close monitoring of serum potassium, and consideration of potassium-lowering therapies when clinically appropriate (17, 35). The DIAMOND trial demonstrated that patiromer reduced hyperkalemia events and enabled greater maintenance of guideline-directed RAAS inhibitor therapy, including MRA use, among patients with HFrEF and a history of hyperkalemia or risk of hyperkalemia (36). Established HFrEF guidelines and randomized clinical trials support continuation of guideline-directed therapies whenever clinically feasible, with appropriate monitoring of serum potassium and renal function (25, 27, 37). These contemporary recommendations reinforce the importance of balancing hyperkalemia risk with the established prognostic benefits of RAAS inhibition and MRA therapy, with treatment modification decisions individualized according to potassium severity, renal function, and overall clinical status (17, 24, 35, 36).

Differences in short-term potassium trajectories were observed according to spironolactone management strategy, suggesting variability in potassium response following different clinical approaches. However, these findings should be interpreted cautiously because spironolactone management decisions were based on clinical judgment rather than random allocation. Patients with more severe clinical presentations, greater renal impairment, higher potassium levels, or overall increased risk may have been more likely to undergo spironolactone discontinuation, resulting in potential confounding by indication. Therefore, the observed potassium changes between management groups cannot be interpreted as demonstrating that continuation or dose reduction of spironolactone directly leads to greater potassium reduction. Given the retrospective observational design, these findings should not be interpreted as establishing specific thresholds for continuation, dose reduction, or discontinuation of spironolactone, but rather emphasize the need for individualized assessment considering potassium severity, renal function, and overall clinical status. Strategies aimed at managing hyperkalemia while preserving guideline-directed RAAS inhibitor and MRA therapy have been emphasized in clinical practice recommendations (37).

Renin–angiotensin–aldosterone system inhibitors, including ACE inhibitors and ARBs, can increase hyperkalemia risk, which may be further influenced when combined with mineralocorticoid receptor antagonists like spironolactone. Consequently, serum potassium and renal function monitoring are recommended in these patients (38). Although hyperkalemia was frequently observed, severity of hyperkalemia was not independently associated with in-hospital mortality in either unadjusted or adjusted analyses. This observation is consistent with real-world evidence suggesting that hyperkalemia itself may not independently determine mortality risk when considered alongside treatment modifications and other clinical characteristics, whereas maintenance of prognostically beneficial RAAS inhibitor therapy remains associated with improved outcomes (30, 37). Similar findings were observed in the EMPHASIS-HF trial, where eplerenone was associated with improved clinical outcomes despite increased hyperkalemia risk (39).

Older age and chronic kidney disease were associated with higher in-hospital mortality in this cohort, consistent with previous evidence demonstrating poorer outcomes among older patients and those with CKD in heart failure populations (40, 41). The mortality findings observed in the present study should be interpreted in the context of a single-center observational cohort and distinguished from treatment effects demonstrated in randomized controlled trials. Nevertheless, large randomized controlled trials have demonstrated beneficial effects of SGLT2 inhibitors in patients with HFrEF. For example, the DAPA-HF trial reported reductions in cardiovascular death and worsening heart failure with dapagliflozin, including decreased all-cause and cardiovascular mortality compared with placebo (42). Similarly, the EMPEROR-Reduced trial demonstrated reductions in cardiovascular death or hospitalization with empagliflozin (43). Furthermore, recent meta-analyses of randomized controlled trials have demonstrated that SGLT2 inhibitors are associated with significant reductions in all-cause mortality, cardiovascular mortality, and heart failure hospitalizations among patients with HFrEF (44). Given the observational design, limited number of mortality events, and potential residual confounding, these associations should be interpreted as hypothesis-generating rather than evidence of causal treatment effects.

GDMT exposure (receipt of ≥3 drug classes) was evaluated as an exploratory factor associated with in-hospital mortality. Although no significant association was observed, the limited number of mortality events and potential residual confounding warrant cautious interpretation, and these findings should be considered exploratory rather than confirmatory. The potential relationship between greater exposure to recommended HFrEF medication classes and clinical outcomes is consistent with previous studies reporting improved outcomes among patients receiving guideline-directed therapy (45, 46). Min et al. reported better clinical outcomes in patients with heart failure with improved ejection fraction who continued GDMT (47). Similarly, Chen et al. reported lower mortality across the heart failure spectrum among patients with greater adherence to guideline-directed therapy (46). Wolfe et al. observed an association between GDMT exposure and reduced mortality among patients with coronary artery disease and HFrEF (48).

From a local clinical audit perspective, the present findings highlight opportunities to evaluate GDMT implementation among hospitalized HFrEF patients within our institution. However, the association between receipt of ≥3 GDMT classes and in-hospital mortality should be interpreted cautiously because of the retrospective observational design, potential confounding by treatment eligibility, and residual differences in disease severity. Although randomized clinical trials and current heart failure guidelines support the use of comprehensive GDMT in eligible patients with HFrEF, the present study provides observational data describing GDMT exposure and short-term in-hospital outcomes within a single-center hospitalized cohort (27, 28, 41, 44–46). Accordingly, these findings should be interpreted cautiously and require confirmation in larger, multicenter studies.

### 4.1 Strengths and limitations

This study provides single-center real-world data from Saudi Arabia on spironolactone management among hospitalized HFrEF patients with hyperkalemia, reflecting routine clinical practice rather than controlled experimental conditions. The cohort was well-characterized with comprehensive data on demographics, comorbidities, echocardiographic parameters, functional status, and laboratory measures, allowing detailed evaluation of treatment patterns, monitoring, and safety outcomes, including hyperkalemia severity and therapy modification. Additionally, spironolactone management was evaluated in the context of contemporary GDMT, including beta-blockers, renin–angiotensin system inhibitors, angiotensin receptor–neprilysin inhibitors, and sodium-glucose co-transporter-2 inhibitors. Exploratory statistical methods, including multivariable regression and Kaplan–Meier survival analyses, were used to evaluate in-hospital outcomes; however, the limited number of mortality events necessitates cautious interpretation of these findings.

Limitations include the retrospective, single-center design, which limits causal inference and generalizability. Therefore, the observed associations between treatment strategies, GDMT exposure, and clinical outcomes should not be interpreted as causal relationships. Furthermore, the study included a highly selected cohort of hospitalized HFrEF patients who were already receiving spironolactone therapy and had documented hyperkalemia (serum potassium >5 mmol/L). Therefore, these findings cannot be used to estimate the incidence of hyperkalemia among all HFrEF patients receiving spironolactone, evaluate overall spironolactone utilization patterns, or determine whether spironolactone itself caused hyperkalemia. Rather, this study represents an evaluation of spironolactone management strategies among patients who developed hyperkalemia during clinical care. Consequently, the findings may not be generalizable to ambulatory (outpatient) patients with HFrEF, patients initiating mineralocorticoid receptor antagonist therapy, or broader heart failure populations managed in different clinical settings.

Data derived from electronic health records may be incomplete or variable, particularly regarding medication dosing, titration, and laboratory monitoring. The short-term potassium assessment over 72 h may have been influenced by biological variation and concurrent inpatient interventions; therefore, changes in serum potassium should be interpreted cautiously. Additionally, potassium changes according to spironolactone management strategy may have been affected by confounding by indication. Patients who discontinued spironolactone had greater clinical complexity and higher-risk characteristics, including renal dysfunction, more severe hyperkalemia, and acute clinical deterioration, which may have influenced both treatment decisions and subsequent potassium trajectories. Therefore, the observed differences in potassium changes between management groups should not be interpreted as evidence of a causal effect of spironolactone continuation, dose reduction, or discontinuation on potassium reduction.

The primary outcome was restricted to in-hospital mortality, and longer-term outcomes, including post-discharge mortality, rehospitalization, and sustained medication management, were not assessed. Only 10 in-hospital mortality events occurred in the cohort, substantially limiting the statistical power of the survival analyses and reducing the precision of the estimated associations. Given the small number of outcome events relative to the number of covariates included in the multivariable Cox regression model, there is an increased risk of model overfitting, whereby the model may fit random variation in the study sample rather than true underlying associations. Consequently, the estimated hazard ratios and confidence intervals may be unstable and should be interpreted with caution. Although multivariable adjustment was performed to account for potential confounders, residual confounding cannot be excluded, particularly with respect to disease severity, renal function, acute illness severity, and treatment selection factors. Therefore, the Cox regression findings should be considered exploratory and hypothesis-generating rather than definitive, and confirmation in larger, multicenter studies with a greater number of outcome events is warranted. Patients receiving broader GDMT or SGLT2 inhibitors may have differed systematically in clinical stability, treatment eligibility, comorbidity profile, and overall severity of illness compared with those receiving fewer therapies. Importantly, receipt of ≥3 GDMT medication classes in this study was based on prescription records and represents a measure of medication class exposure rather than a comprehensive assessment of GDMT adherence, optimization, or treatment quality. This approach does not capture target dose achievement, dose titration, treatment duration, treatment persistence, contraindication-adjusted eligibility, or patient-level medication adherence. Therefore, GDMT exposure may have been misclassified, and these findings should be interpreted as reflecting prescribed medication class exposure rather than the effectiveness or quality of GDMT implementation.

### 4.2 Clinical implications

In patients with HFrEF receiving spironolactone, regular monitoring of serum potassium and renal function is essential to guide individualized dose adjustments and minimize the risk of hyperkalemia while preserving guideline-directed medical therapy whenever clinically appropriate. In this cohort, spironolactone continuation, dose reduction, and discontinuation reflected real-world clinical decision-making following hyperkalemia detection and should be interpreted in the context of patients' overall clinical status, renal function, and severity of illness. Receipt of ≥3 GDMT medication classes was associated with a lower adjusted hazard estimate for in-hospital mortality; however, this association was not statistically significant and may have been influenced by treatment selection factors and residual confounding. Therefore, these findings should not be interpreted as demonstrating a causal mortality benefit of broader GDMT exposure in this selected cohort. Overall, the findings highlight the importance of individualized management strategies for hyperkalemia in hospitalized patients with HFrEF, with careful consideration of potassium levels, renal function, treatment eligibility, and the potential need for strategies that allow continuation of evidence-based therapies when clinically feasible.

## 5 Conclusion

In this retrospective cohort of hospitalized patients with HFrEF and hyperkalemia (serum potassium >5 mmol/L), most patients had mild-to-moderate potassium elevations, and spironolactone management most commonly involved dose reduction or discontinuation. This study describes contemporary real-world spironolactone management patterns and associated short-term clinical outcomes following hyperkalemia in hospitalized patients with HFrEF. In the exploratory multivariable analyses, older age and chronic kidney disease were associated with higher in-hospital mortality. Receipt of ≥3 GDMT classes was associated with a lower hazard estimate for in-hospital mortality; however, this association did not reach statistical significance. Because of the retrospective observational design, the limited number of mortality events, and the potential for residual confounding, these findings represent associations rather than causal effects and should be interpreted as exploratory and hypothesis-generating. Overall, these findings provide insight into current clinical practice and may inform future prospective studies evaluating spironolactone management strategies for hospitalized patients with HFrEF who develop hyperkalemia.

## Data Availability

The original contributions presented in the study are included in the article/Supplementary material, further inquiries can be directed to the corresponding author.
