# Supplementary material for: Patterns of spironolactone management following hyperkalemia among hospitalized patients with heart failure and reduced ejection fraction: a retrospective single-center study
Source: Front Med (Lausanne). 2026 Aug 24;13:1870470. doi: 10.3389/fmed.2026.1870470 (PMC13547031; doi:10.3389/fmed.2026.1870470)
Supplement: Supplementary file 1 [file Table_1.docx]

**Supplementary Table 1**

| **Variable** | **Mild–moderate (n=117)** | **Severe (n=19)** | **P-value** |
| --- | --- | --- | --- |
| Age, years | 57.90 ± 12.41 | 60.85± 13.73 | 0.34 |
| Male sex | 98 (83.76) | 16 (84.21) | 1.000 |
| CKD | 21 (17.95) | 7 (36.84) | 0.078 |
| Creatinine, mg/dL | 1.28 ± 0.42 | 1.62 ± 0.61 | 0.008 |
| SGLT2 inhibitor use | 53 (45.30) | 7 (36.84) | 0.092 |
| GDMT ≥3 classes | 49 (41.88) | 5 (26.31) | 0.24 |

**Supplementary Table 2**

**Baseline Clinical Covariates, Renal Parameters, and Management Characteristics According to Spironolactone Management Strategy in Hospitalized HFrEF Patients With Hyperkalemia**

| **Variable** | **Continued (n=18)** | **Dose Reduced (n=39)** | **Discontinued (n=79)** | **P-value** |
| --- | --- | --- | --- | --- |
| eGFR (mL/min/1.73m²) | 82.40 ± 24.30 | 74.80 ± 27.10 | 61.20 ± 28.50 | 0.008 |
| eGFR ≥60, n (%) | 15 (83.33) | 28 (71.79) | 39 (49.37) | 0.011 |
| eGFR 30–59, n (%) | 3 (16.67) | 9 (23.08) | 29 (36.71) | 0.094 |
| eGFR <30, n (%) | 0 (0.00) | 2 (5.13) | 11 (13.92) | 0.082 |
| AKI at admission, n (%) | 1 (5.56) | 5 (12.82) | 20 (25.32) | 0.041 |
| Serum creatinine (mg/dL) | 1.12 ± 0.30 | 1.25 ± 0.38 | 1.47 ± 0.51 | 0.010 |
| Baseline potassium (mmol/L) | 5.25 ± 0.30 | 5.37 ± 0.32 | 5.27 ± 0.28 | 0.250 |
| Mild–moderate hyperkalemia, n (%) | 18 (100.00) | 37 (94.87) | 62 (78.48) | <0.001 |
| Severe hyperkalemia, n (%) | 0 (0.00) | 2 (5.13) | 17 (21.52) | <0.001 |
| Spironolactone dose (mg/day) | 25.00 ± 0.00 | 30.10 ± 11.20 | 36.70 ± 15.40 | 0.004 |
| Spironolactone 25 mg/day, n (%) | 18 (100.00) | 28 (71.79) | 42 (53.16) | <0.001 |
| Spironolactone 50 mg/day, n (%) | 0 (0.00) | 11 (28.21) | 37 (46.84) | <0.001 |
| ACEi use, n (%) | 9 (50.00) | 18 (46.15) | 29 (36.71) | 0.410 |
| ARB use, n (%) | 4 (22.22) | 8 (20.51) | 16 (20.25) | 0.980 |
| ARNI use, n (%) | 8 (44.44) | 13 (33.33) | 14 (17.72) | 0.037 |
| Loop diuretic use, n (%) | 14 (77.78) | 33 (84.62) | 72 (91.14) | 0.220 |
| Thiazide diuretic use, n (%) | 2 (11.11) | 6 (15.38) | 15 (18.99) | 0.730 |
| Potassium binder use, n (%) | 4 (22.22) | 15 (38.46) | 42 (53.16) | 0.018 |
| Insulin-dextrose therapy, n (%) | 10 (55.56) | 29 (74.36) | 68 (86.08) | 0.002 |
| Sodium bicarbonate therapy, n (%) | 1 (5.56) | 4 (10.26) | 12 (15.19) | 0.410 |
| Systolic BP (mmHg) | 118.50 ± 14.60 | 114.20 ± 16.10 | 108.90 ± 17.80 | 0.046 |
| Diastolic BP (mmHg) | 72.40 ± 8.90 | 69.10 ± 9.20 | 66.80 ± 10.40 | 0.120 |
| ICU admission, n (%) | 1 (5.56) | 5 (12.82) | 18 (22.78) | 0.087 |
| NYHA III–IV, n (%) | 10 (55.56) | 25 (64.10) | 56 (70.88) | 0.096 |
| LVEF (%) | 26.80 ± 7.16 | 25.43 ± 7.97 | 24.70 ± 8.02 | 0.520 |
| Acute decompensated HF, n (%) | 8 (44.44) | 22 (56.41) | 56 (70.89) | 0.030 |

**Supplementary Table 3**

**Baseline Clinical Characteristics According to Spironolactone Management Strategy Among Hospitalized Patients WithHFrEF and Hyperkalemia**

| **Variable** | **Continued (n=18)** | **Dose Reduced (n=39)** | **Discontinued (n=79)** | **P-value** |
| --- | --- | --- | --- | --- |
| Age, years | 55.27 ± 11.31 | 57.87 ± 12.50 | 60.16 ± 13.24 | 0.18 |
| Male sex, n (%) | 15 (83.33) | 33 (84.62) | 66 (83.54) | 0.98 |
| LVEF, % | 26.80 ± 7.16 | 25.43 ± 7.97 | 24.70 ± 8.02 | 0.52 |
| NYHA III–IV, n (%) | 10 (55.56) | 25 (64.10) | 56 (70.88) | 0.096 |
| Chronic kidney disease, n (%) | 2 (11.11) | 5 (12.82) | 21 (26.58) | 0.065 |
| Serum creatinine, mg/dL | 1.12 ± 0.30 | 1.25 ± 0.38 | 1.47 ± 0.51 | 0.01 |
| Baseline potassium, mmol/L | 5.25 ± 0.30 | 5.37 ± 0.32 | 5.27 ± 0.28 | 0.25 |
| Severe hyperkalemia, n (%) | 0 (0.00) | 2 (5.13) | 17 (21.51) | <0.001 |
| SGLT2 inhibitor use, n (%) | 10 (55.56) | 18 (46.15) | 32 (40.51) | 0.36 |
| Receipt of ≥3 GDMT classes, n (%) | 8 (42.10) | 29 (74.36) | 17 (21.51) | 0.071 |
